# (4*R*)-3-Hy­droxy-7-isopropyl-4-methyl-5,6-di­hydro­benzo­furan-2(4*H*)-one

**DOI:** 10.1107/S1600536814014524

**Published:** 2014-06-25

**Authors:** Frank W. Heinemann, Alberto Herrera, Giuseppe Agrifoglio, Romano Dorta, Jesús Pastrán

**Affiliations:** a Institut für Anorganische Chemie, Universität Erlangen-Nürnberg, Egerlandstrasse 1, D-91058 Erlangen, Germany; b Departamento de Química, Universidad Simón Bolívar, Apartado 89000, Caracas 1020-A, Venezuela

**Keywords:** crystal structure

## Abstract

In the title compound, alternatively called α-hy­droxy-γ-alkyl­idenebutenolide, C_12_H_16_O_3_, two independent mol­ecules (*A* and *B*) crystallize in the asymmetric unit in each of which the 5,6-di­hydro­benzo ring has an envelope conformation. The torsion angle along the butadiene chain in the γ-alkyl­idenebutenolide core is −177.9 (2)° for mol­ecule *A* and 179.9 (2)° for mol­ecule *B*. In the crystal, O—H⋯O hydrogen bonds between hy­droxyl and carbonyl groups of adjacent independent mol­ecules form dimers with *R* ^2^_2_(10) loops.

## Related literature   

For background to butenolides and their pharmacological activity, see: Rao (1964 ▶); Ma *et al.* (1999 ▶). For the synthesis of γ-alkyl­idenebutenolides, see: Park *et al.* (2012 ▶); Almeida *et al.* (2010 ▶); Xu *et al.* (2007 ▶); Langer *et al.* (2000 ▶, 2001 ▶). For related structures, see: Schneider & Viljoen (1997 ▶); Langer & Saleh (2000 ▶). For standard bond lengths, see: Allen *et al.* (1987 ▶) and for puckering parameters, see: Cremer & Pople (1975 ▶).
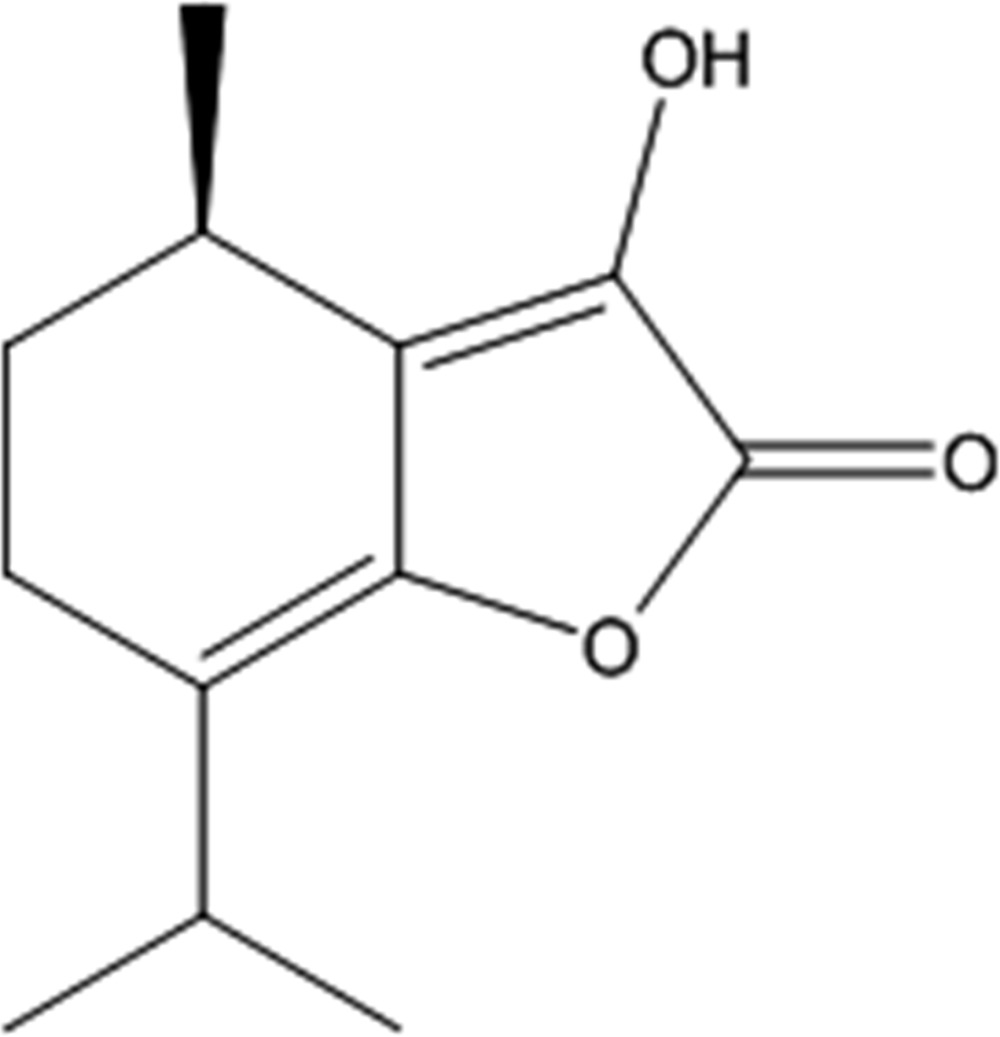


## Experimental   

### 

#### Crystal data  

C_12_H_16_O_3_
*M* *_r_* = 208.25
Monoclinic, 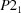

*a* = 9.0437 (3) Å
*b* = 13.2792 (6) Å
*c* = 9.8199 (5) Å
β = 104.694 (3)°
*V* = 1140.73 (9) Å^3^
*Z* = 4
Mo *K*α radiation
μ = 0.09 mm^−1^
*T* = 150 K
0.55 × 0.20 × 0.20 mm

#### Data collection  

Bruker–Nonius KappaCCD diffractometer
Absorption correction: multi-scan (*SADABS*; Bruker, 2002 ▶) *T*_min_ = 0.682, *T*_max_ = 0.746
37926 measured reflections
2821 independent reflections
2564 reflections with *I* > 2σ(*I*)
*R*_int_ = 0.042

#### Refinement  

*R*[*F* ^2^ > 2σ(*F* ^2^)] = 0.037
*wR*(*F* ^2^) = 0.103
*S* = 1.09
2821 reflections
283 parameters
1 restraint
H atoms treated by a mixture of independent and constrained refinement
Δρ_max_ = 0.26 e Å^−3^
Δρ_min_ = −0.22 e Å^−3^

### 

Data collection: *COLLECT* (Bruker–Nonius, 2002 ▶); cell refinement: *EVALCCD* (Duisenberg *et al.*, 2003 ▶); data reduction: *EVALCCD* (Duisenberg *et al.*, 2003 ▶); program(s) used to solve structure: *SHELXTL* (Sheldrick, 2008 ▶); program(s) used to refine structure: *SHELXTL*; molecular graphics: *SHELXTL*; software used to prepare material for publication: *SHELXTL*.

## Supplementary Material

Crystal structure: contains datablock(s) I. DOI: 10.1107/S1600536814014524/jj2187sup1.cif

Structure factors: contains datablock(s) I. DOI: 10.1107/S1600536814014524/jj2187Isup2.hkl

Supporting information file. DOI: 10.1107/S1600536814014524/jj2187Isup3.cml

CCDC reference: 1009320

Additional supporting information:  crystallographic information; 3D view; checkCIF report

## Figures and Tables

**Table 1:** Hydrogen-bond geometry (Å, °)

*D*—H⋯*A*	*D*—H	H⋯*A*	*D*⋯*A*	*D*—H⋯*A*
O3—H3⋯O5^i^	0.87 (3)	1.81 (3)	2.627 (2)	157 (3)
O6—H6⋯O2^ii^	0.84 (3)	1.93 (3)	2.727 (2)	156 (3)

Symmetry codes: (i) 
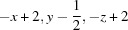
; (ii) 
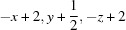
.

**Fig. 1.**
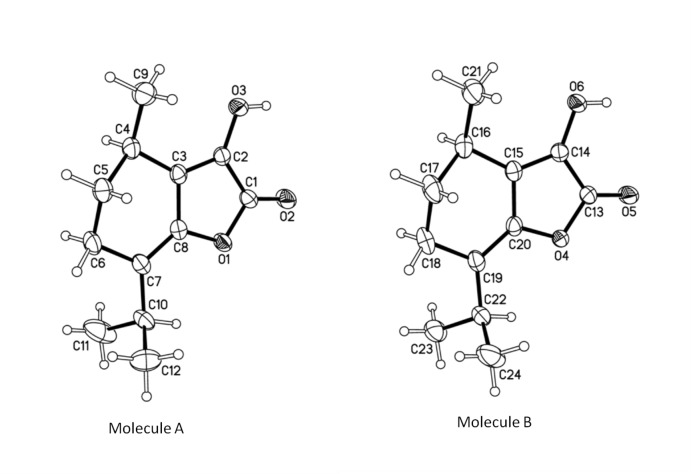
View of independent molecules A and B of the title compound, C12H16O3, showing the atom-labelling scheme. Displacement ellipsoids are drawn at the 50% probability level.

**Fig. 2.**
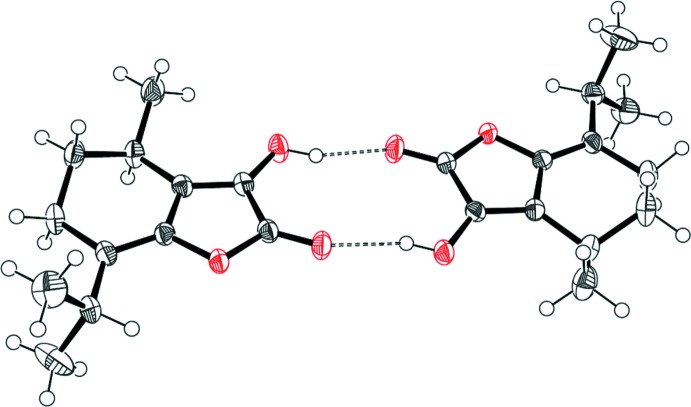
Inversion dimer formed in the molecular packing of the title compound. Dashed lines indicate intermolecular O–H···O hydrogen bonds between hydroxyl and the carbonyl groups of neighboring molecules.

## supplementary crystallographic information

### S1. Introduction

Butenolides are an important class of organic compounds present in natural products that have been studied for over 50 years (Rao, 1964). Most of them exhibit inter­esting pharmacological activities, such as anti­bacterial, anti­cancer, anti­biotic and phospho­lipase A2 inhibition activity (Ma *et al*, 1999). During the last decades γ-alkyl­idenebutenolides have been considered as attractive synthetic targets due to their structural diversity and biological properties. As a result, several synthetic procedures have been developed for the preparation of these substances (Langer, *et al.*, 2000; Langer, *et al.*, 2001; Xu, *et al.*, 2007; Almeida, *et al*., 2010; Park, *et al.*, 2012). Also, α-Hy­droxy-γ-alkyl­idenebutenolides are particularly suitable building blocks for analogues of pharmacologically relevant natural products (Langer & Saleh, 2000). Herein, we report the crystal structure of a bicyclic α-hy­droxy-γ-alkyl­idenebutenolide based on *l*-Menthone, an inexpensive and accessible reagent from the chiral pool, which is also an important structural motif found in natural products. To the best of our knowledge, there is only one report on the preparation of a similar γ-alkyl­idenebutenolide, but no structural data were presented (Schneider & Viljoen, 1997).

### S2.1. Synthesis and crystallization

Sodium hydride (60% dispersion in mineral oil, 2.44 g, 0.061 mmol) was stirred for 15 minutes in 200 mL of freshly distilled THF. Then a mixture of *l*-Menthone (7.71 g, 0.050 mmol) and di-ethyl oxalate (3.36 g, 0.023 mmol) in 100 mL of THF was added drop by drop. The resulting mixture was heated to reflux for 2 days. After this time, the solvent was removed by rotary evaporation. The crude reaction product was added to an ice-hydro­chloric acid (1M) mixture and extracted with chloro­form (3 x 50 mL). The organic layer was dried with MgSO_4_, filtered and the solvent was removed under vacuum to afford orange oil, which was purified by Kugelrohr distillation (413 °K, 5 x 10^-2^ mbar, bulbs cooled with dry ice), to obtain the desired product as a yellow oil that solidifies (1.95 g, 41%). Suitable crystals for X-ray diffraction analysis were obtained by slow diffusion of hexane into a saturated solution of the compound in di­chloro­methane cooled at 263 °K for 3 days. Elemental analysis calculated for C_12_H_16_O_3_×1/3H_2_O: C, 67.27 %, H 7.84 %. Found: C, 67.38 %, H 7.70 %.

### S2.2. Refinement

The positions of the two oxygen bound hydrogen atoms H3 and H6 were taken from a difference fourier synthesis and their positional parameters were refined. All other H atoms were included in calculated positions (C–H = 0.93 Å for aromatic H, C–H = 0.96 Å for methyl H, C–H = 0.98 Å for methyl­ene H, and C–H = 1.00 Å for tertiary H), and refined using a riding model with *U*_iso_(H) = 1.2 Ueq or *U*_iso_ (H) = 1.5 Ueq (for methyl groups) of the carrier atom.

### S3. Results and discussion

In the title compound,C_12_H_16_O_3_, two independent molecules (A and B) crystallize in the asymmetric unit (Fig. 1). The 5,6-di­hydro­benzo ring has an envelope conformation (puckering parameters Q, θ, and φ = 0.458 (2)Å, 126.4 (2)° and 295.8 (3)°, respectively; (Cremer & Pople, 1975)). The torsion angles along the butadiene chain in the γ-alkyl­idenebutenolide core are -177.9 (2)° for molecule A and 179.9 (2)° for molecule B. Bond lengths are in normal ranges (Allen *et al.*, 1987). In the crystal O–H···O hydrogen bonds between hydroxyl and carbonyl groups of adjacent independent molecules form inversion dimers (Fig. 2).

### Crystal data

**Table:**

C_12_H_16_O_3_	*F*(000) = 448
*M**_r_* = 208.25	*D*_x_ = 1.213 Mg m^−^^3^
Monoclinic, *P*2_1_	Mo *K*α radiation, λ = 0.71073 Å
Hall symbol: P 2yb	Cell parameters from 96 reflections
*a* = 9.0437 (3) Å	θ = 6.0–20.0°
*b* = 13.2792 (6) Å	µ = 0.09 mm^−^^1^
*c* = 9.8199 (5) Å	*T* = 150 K
β = 104.694 (3)°	Block, colorless
*V* = 1140.73 (9) Å^3^	0.55 × 0.20 × 0.20 mm
*Z* = 4	

### Data collection

**Table:**

Bruker–Nonius KappaCCD diffractometer	2821 independent reflections
Radiation source: fine-focus sealed tube	2564 reflections with *I* > 2σ(*I*)
Graphite monochromator	*R*_int_ = 0.042
Detector resolution: 9 pixels mm^-1^	θ_max_ = 27.9°, θ_min_ = 3.1°
φ– and ω–rotations with 2.00 ° and 60 sec per frame scans	*h* = −11→11
Absorption correction: multi-scan (*SADABS*; Bruker, 2002)	*k* = −17→17
*T*_min_ = 0.682, *T*_max_ = 0.746	*l* = −12→12
37926 measured reflections	

### Refinement

**Table:**

Refinement on *F*^2^	Primary atom site location: structure-invariant direct methods
Least-squares matrix: full	Secondary atom site location: difference Fourier map
*R*[*F*^2^ > 2σ(*F*^2^)] = 0.037	Hydrogen site location: inferred from neighbouring sites
*wR*(*F*^2^) = 0.103	H atoms treated by a mixture of independent and constrained refinement
*S* = 1.09	*w* = 1/[σ^2^(*F*_o_^2^) + (0.064*P*)^2^ + 0.1458*P*] where *P* = (*F*_o_^2^ + 2*F*_c_^2^)/3
2821 reflections	(Δ/σ)_max_ < 0.001
283 parameters	Δρ_max_ = 0.26 e Å^−^^3^
1 restraint	Δρ_min_ = −0.22 e Å^−^^3^

### Special details

**Table:**

Experimental. ^1^H NMR (400 MHz, CDCl_3_) (δ, p.p.m..): 1.01–1.04 (t, 6H), 1.29–1.31 (d, 3H), 1.43–1.51 (m, 1H), 1.81–1.88 (m, 1H), 2.14–2.21 (m, 1H), 2.26–2.33 (m, 1H), 2.74–2.83 (m, 1H), 3.04–3.11 (m, 1H). ^13^C{1H} NMR (101 MHz, CDCl_3_) (δ, p.p.m..): 17.65, 20.23, 20.40, 21.95, 27.89, 28.25, 31.22, 128.25, 128.75, 135.33, 140.89, 167.80. [α]_D_^20^ = +4.32 (c 0.018, CH_3_OH)
Geometry. All e.s.d.'s (except the e.s.d. in the dihedral angle between two l.s. planes) are estimated using the full covariance matrix. The cell e.s.d.'s are taken into account individually in the estimation of e.s.d.'s in distances, angles and torsion angles; correlations between e.s.d.'s in cell parameters are only used when they are defined by crystal symmetry. An approximate (isotropic) treatment of cell e.s.d.'s is used for estimating e.s.d.'s involving l.s. planes.
Refinement. Refinement of *F*^2^ against ALL reflections. The weighted *R*-factor *wR* and goodness of fit *S* are based on *F*^2^, conventional *R*-factors *R* are based on *F*, with *F* set to zero for negative *F*^2^. The threshold expression of *F*^2^ > σ(*F*^2^) is used only for calculating *R*-factors(gt) *etc*. and is not relevant to the choice of reflections for refinement. *R*-factors based on *F*^2^ are statistically about twice as large as those based on *F*, and *R*- factors based on ALL data will be even larger.

### Fractional atomic coordinates and isotropic or equivalent isotropic displacement parameters (Å^2^)

**Table:**

	*x*	*y*	*z*	*U*_iso_*/*U*_eq_	
O1	0.61403 (15)	0.10519 (12)	1.04435 (15)	0.0267 (3)	
O2	0.81828 (17)	0.02807 (13)	1.18582 (17)	0.0340 (4)	
O3	0.98766 (16)	0.21407 (13)	1.14537 (16)	0.0311 (3)	
H3	1.033 (3)	0.165 (3)	1.197 (3)	0.047*	
O4	0.59682 (16)	0.52380 (12)	0.55678 (16)	0.0293 (3)	
O5	0.80720 (19)	0.59606 (14)	0.6939 (2)	0.0474 (5)	
O6	0.95840 (16)	0.40045 (12)	0.67379 (16)	0.0293 (3)	
H6	1.005 (3)	0.450 (3)	0.719 (3)	0.044*	
C1	0.7648 (2)	0.10095 (17)	1.1155 (2)	0.0247 (4)	
C2	0.8387 (2)	0.19425 (16)	1.08940 (19)	0.0230 (4)	
C3	0.7337 (2)	0.25226 (16)	1.00204 (18)	0.0231 (4)	
C4	0.7400 (2)	0.35151 (18)	0.9315 (2)	0.0299 (5)	
H4A	0.7655	0.3382	0.8399	0.036*	
C5	0.5796 (3)	0.39841 (18)	0.8990 (2)	0.0339 (5)	
H5A	0.5559	0.4175	0.9886	0.041*	
H5B	0.5791	0.4606	0.8433	0.041*	
C6	0.4551 (2)	0.32752 (19)	0.8177 (2)	0.0329 (5)	
H6B	0.3540	0.3592	0.8089	0.039*	
H6C	0.4685	0.3179	0.7215	0.039*	
C7	0.4576 (2)	0.22601 (17)	0.8872 (2)	0.0256 (4)	
C8	0.5915 (2)	0.19749 (16)	0.97278 (19)	0.0234 (4)	
C9	0.8623 (3)	0.4221 (2)	1.0174 (3)	0.0431 (6)	
H9A	0.9632	0.3910	1.0303	0.065*	
H9B	0.8424	0.4342	1.1096	0.065*	
H9C	0.8596	0.4862	0.9674	0.065*	
C10	0.3163 (2)	0.16088 (18)	0.8569 (2)	0.0316 (5)	
H10A	0.3435	0.0952	0.9068	0.038*	
C11	0.2613 (3)	0.1398 (3)	0.6991 (3)	0.0610 (9)	
H11A	0.3450	0.1104	0.6655	0.091*	
H11B	0.2284	0.2029	0.6488	0.091*	
H11C	0.1752	0.0926	0.6817	0.091*	
C12	0.1891 (3)	0.2093 (3)	0.9104 (3)	0.0492 (7)	
H12A	0.2233	0.2179	1.0127	0.074*	
H12B	0.0984	0.1661	0.8872	0.074*	
H12C	0.1639	0.2753	0.8656	0.074*	
C13	0.7478 (2)	0.52326 (17)	0.6280 (2)	0.0289 (4)	
C14	0.8127 (2)	0.42486 (16)	0.60962 (19)	0.0233 (4)	
C15	0.7014 (2)	0.36769 (15)	0.52805 (19)	0.0227 (4)	
C16	0.6920 (2)	0.26074 (17)	0.4779 (2)	0.0297 (4)	
H16A	0.6593	0.2184	0.5492	0.036*	
C17	0.5661 (3)	0.2546 (2)	0.3396 (2)	0.0382 (5)	
H17A	0.6003	0.2913	0.2653	0.046*	
H17B	0.5507	0.1832	0.3104	0.046*	
C18	0.4140 (3)	0.2984 (2)	0.3514 (3)	0.0362 (5)	
H18A	0.3426	0.3000	0.2566	0.043*	
H18B	0.3697	0.2533	0.4110	0.043*	
C19	0.4274 (2)	0.40341 (17)	0.4132 (2)	0.0264 (4)	
C20	0.5657 (2)	0.42904 (16)	0.49253 (19)	0.0241 (4)	
C21	0.8449 (3)	0.2199 (2)	0.4629 (3)	0.0409 (6)	
H21A	0.9211	0.2249	0.5535	0.061*	
H21B	0.8789	0.2594	0.3921	0.061*	
H21C	0.8329	0.1492	0.4334	0.061*	
C22	0.2912 (2)	0.47316 (19)	0.3872 (3)	0.0343 (5)	
H22A	0.3169	0.5299	0.4561	0.041*	
C23	0.1499 (3)	0.4196 (2)	0.4103 (3)	0.0407 (6)	
H23A	0.1726	0.3927	0.5063	0.061*	
H23B	0.1214	0.3643	0.3427	0.061*	
H23C	0.0650	0.4675	0.3968	0.061*	
C24	0.2587 (3)	0.5178 (3)	0.2387 (3)	0.0612 (9)	
H24A	0.3492	0.5541	0.2275	0.092*	
H24B	0.1721	0.5644	0.2246	0.092*	
H24C	0.2340	0.4634	0.1691	0.092*	

### Atomic displacement parameters (Å^2^)

**Table:**

	*U*^11^	*U*^22^	*U*^33^	*U*^12^	*U*^13^	*U*^23^
O1	0.0214 (7)	0.0257 (8)	0.0292 (7)	0.0018 (6)	−0.0005 (6)	0.0038 (6)
O2	0.0256 (7)	0.0287 (8)	0.0423 (9)	0.0028 (7)	−0.0013 (6)	0.0112 (7)
O3	0.0190 (7)	0.0347 (9)	0.0368 (8)	0.0006 (6)	0.0016 (6)	0.0113 (7)
O4	0.0244 (7)	0.0211 (7)	0.0349 (8)	−0.0017 (6)	−0.0065 (6)	−0.0013 (6)
O5	0.0308 (9)	0.0260 (9)	0.0684 (12)	0.0010 (7)	−0.0191 (8)	−0.0123 (9)
O6	0.0213 (7)	0.0269 (8)	0.0351 (8)	0.0001 (6)	−0.0016 (6)	−0.0064 (6)
C1	0.0210 (9)	0.0280 (10)	0.0237 (9)	0.0032 (8)	0.0029 (7)	0.0000 (8)
C2	0.0213 (9)	0.0276 (11)	0.0206 (8)	0.0032 (8)	0.0061 (7)	0.0020 (8)
C3	0.0243 (9)	0.0277 (10)	0.0175 (8)	0.0028 (8)	0.0058 (7)	−0.0003 (8)
C4	0.0270 (10)	0.0324 (11)	0.0292 (10)	0.0047 (9)	0.0050 (8)	0.0098 (9)
C5	0.0321 (11)	0.0286 (11)	0.0384 (11)	0.0061 (9)	0.0040 (9)	0.0106 (9)
C6	0.0275 (10)	0.0381 (13)	0.0307 (11)	0.0103 (9)	0.0030 (9)	0.0112 (9)
C7	0.0227 (9)	0.0322 (11)	0.0208 (8)	0.0051 (8)	0.0033 (7)	−0.0011 (8)
C8	0.0254 (9)	0.0241 (10)	0.0200 (8)	0.0036 (8)	0.0048 (7)	0.0009 (8)
C9	0.0368 (12)	0.0304 (12)	0.0555 (15)	−0.0035 (10)	−0.0005 (11)	0.0128 (11)
C10	0.0215 (9)	0.0337 (12)	0.0350 (11)	0.0053 (9)	−0.0013 (8)	0.0019 (9)
C11	0.0360 (13)	0.093 (3)	0.0480 (16)	−0.0079 (15)	−0.0006 (11)	−0.0274 (17)
C12	0.0327 (12)	0.0598 (18)	0.0610 (16)	0.0009 (12)	0.0231 (12)	−0.0029 (15)
C13	0.0255 (9)	0.0239 (10)	0.0310 (10)	−0.0035 (8)	−0.0046 (8)	0.0009 (8)
C14	0.0244 (9)	0.0244 (10)	0.0193 (8)	−0.0024 (8)	0.0025 (7)	0.0026 (7)
C15	0.0245 (9)	0.0244 (10)	0.0196 (8)	−0.0048 (7)	0.0066 (7)	−0.0006 (7)
C16	0.0314 (10)	0.0267 (10)	0.0332 (10)	−0.0078 (9)	0.0121 (8)	−0.0069 (9)
C17	0.0335 (11)	0.0433 (13)	0.0375 (11)	−0.0107 (11)	0.0085 (9)	−0.0199 (11)
C18	0.0307 (11)	0.0395 (13)	0.0363 (12)	−0.0151 (10)	0.0046 (9)	−0.0096 (10)
C19	0.0257 (10)	0.0293 (11)	0.0224 (9)	−0.0088 (9)	0.0030 (8)	0.0026 (8)
C20	0.0269 (9)	0.0228 (10)	0.0210 (9)	−0.0060 (8)	0.0032 (7)	0.0034 (8)
C21	0.0327 (11)	0.0388 (13)	0.0531 (14)	−0.0034 (10)	0.0140 (10)	−0.0181 (11)
C22	0.0248 (10)	0.0339 (13)	0.0382 (12)	−0.0064 (9)	−0.0031 (9)	0.0060 (10)
C23	0.0274 (11)	0.0453 (15)	0.0488 (14)	−0.0038 (10)	0.0087 (10)	0.0080 (12)
C24	0.0353 (13)	0.075 (2)	0.0624 (18)	−0.0123 (14)	−0.0078 (12)	0.0402 (17)

### Geometric parameters (Å, º)

**Table:**

O1—C1	1.367 (2)	C11—H11B	0.9800
O1—C8	1.402 (3)	C11—H11C	0.9800
O2—C1	1.216 (3)	C12—H12A	0.9800
O3—C2	1.346 (2)	C12—H12B	0.9800
O3—H3	0.87 (3)	C12—H12C	0.9800
O4—C13	1.367 (2)	C13—C14	1.462 (3)
O4—C20	1.404 (3)	C14—C15	1.350 (3)
O5—C13	1.210 (3)	C15—C20	1.440 (3)
O6—C14	1.348 (2)	C15—C16	1.499 (3)
O6—H6	0.84 (3)	C16—C21	1.527 (3)
C1—C2	1.461 (3)	C16—C17	1.538 (3)
C2—C3	1.348 (3)	C16—H16A	1.0000
C3—C8	1.442 (3)	C17—C18	1.524 (3)
C3—C4	1.497 (3)	C17—H17A	0.9900
C4—C9	1.529 (3)	C17—H17B	0.9900
C4—C5	1.536 (3)	C18—C19	1.514 (3)
C4—H4A	1.0000	C18—H18A	0.9900
C5—C6	1.528 (3)	C18—H18B	0.9900
C5—H5A	0.9900	C19—C20	1.339 (3)
C5—H5B	0.9900	C19—C22	1.510 (3)
C6—C7	1.509 (3)	C21—H21A	0.9800
C6—H6B	0.9900	C21—H21B	0.9800
C6—H6C	0.9900	C21—H21C	0.9800
C7—C8	1.341 (3)	C22—C23	1.530 (3)
C7—C10	1.509 (3)	C22—C24	1.531 (4)
C9—H9A	0.9800	C22—H22A	1.0000
C9—H9B	0.9800	C23—H23A	0.9800
C9—H9C	0.9800	C23—H23B	0.9800
C10—C12	1.523 (3)	C23—H23C	0.9800
C10—C11	1.529 (4)	C24—H24A	0.9800
C10—H10A	1.0000	C24—H24B	0.9800
C11—H11A	0.9800	C24—H24C	0.9800
			
C1—O1—C8	106.96 (16)	H12A—C12—H12C	109.5
C2—O3—H3	112 (2)	H12B—C12—H12C	109.5
C13—O4—C20	106.58 (16)	O5—C13—O4	121.3 (2)
C14—O6—H6	111 (2)	O5—C13—C14	129.96 (19)
O2—C1—O1	121.6 (2)	O4—C13—C14	108.76 (18)
O2—C1—C2	129.88 (19)	O6—C14—C15	129.5 (2)
O1—C1—C2	108.47 (17)	O6—C14—C13	122.17 (18)
O3—C2—C3	128.2 (2)	C15—C14—C13	108.26 (18)
O3—C2—C1	123.31 (18)	C14—C15—C20	106.68 (18)
C3—C2—C1	108.47 (18)	C14—C15—C16	134.4 (2)
C2—C3—C8	106.69 (19)	C20—C15—C16	118.89 (17)
C2—C3—C4	134.0 (2)	C15—C16—C21	113.05 (18)
C8—C3—C4	119.27 (17)	C15—C16—C17	107.96 (19)
C3—C4—C9	113.05 (18)	C21—C16—C17	112.45 (19)
C3—C4—C5	107.98 (17)	C15—C16—H16A	107.7
C9—C4—C5	112.3 (2)	C21—C16—H16A	107.7
C3—C4—H4A	107.8	C17—C16—H16A	107.7
C9—C4—H4A	107.8	C18—C17—C16	113.12 (19)
C5—C4—H4A	107.8	C18—C17—H17A	109.0
C6—C5—C4	113.0 (2)	C16—C17—H17A	109.0
C6—C5—H5A	109.0	C18—C17—H17B	109.0
C4—C5—H5A	109.0	C16—C17—H17B	109.0
C6—C5—H5B	109.0	H17A—C17—H17B	107.8
C4—C5—H5B	109.0	C19—C18—C17	113.62 (18)
H5A—C5—H5B	107.8	C19—C18—H18A	108.8
C7—C6—C5	112.91 (17)	C17—C18—H18A	108.8
C7—C6—H6B	109.0	C19—C18—H18B	108.8
C5—C6—H6B	109.0	C17—C18—H18B	108.8
C7—C6—H6C	109.0	H18A—C18—H18B	107.7
C5—C6—H6C	109.0	C20—C19—C22	123.0 (2)
H6B—C6—H6C	107.8	C20—C19—C18	115.8 (2)
C8—C7—C10	123.1 (2)	C22—C19—C18	121.25 (18)
C8—C7—C6	116.31 (19)	C19—C20—O4	122.70 (19)
C10—C7—C6	120.55 (17)	C19—C20—C15	127.6 (2)
C7—C8—O1	123.65 (19)	O4—C20—C15	109.70 (16)
C7—C8—C3	126.9 (2)	C16—C21—H21A	109.5
O1—C8—C3	109.41 (16)	C16—C21—H21B	109.5
C4—C9—H9A	109.5	H21A—C21—H21B	109.5
C4—C9—H9B	109.5	C16—C21—H21C	109.5
H9A—C9—H9B	109.5	H21A—C21—H21C	109.5
C4—C9—H9C	109.5	H21B—C21—H21C	109.5
H9A—C9—H9C	109.5	C19—C22—C23	111.5 (2)
H9B—C9—H9C	109.5	C19—C22—C24	110.5 (2)
C7—C10—C12	111.4 (2)	C23—C22—C24	110.81 (19)
C7—C10—C11	110.3 (2)	C19—C22—H22A	108.0
C12—C10—C11	110.3 (2)	C23—C22—H22A	108.0
C7—C10—H10A	108.2	C24—C22—H22A	108.0
C12—C10—H10A	108.2	C22—C23—H23A	109.5
C11—C10—H10A	108.2	C22—C23—H23B	109.5
C10—C11—H11A	109.5	H23A—C23—H23B	109.5
C10—C11—H11B	109.5	C22—C23—H23C	109.5
H11A—C11—H11B	109.5	H23A—C23—H23C	109.5
C10—C11—H11C	109.5	H23B—C23—H23C	109.5
H11A—C11—H11C	109.5	C22—C24—H24A	109.5
H11B—C11—H11C	109.5	C22—C24—H24B	109.5
C10—C12—H12A	109.5	H24A—C24—H24B	109.5
C10—C12—H12B	109.5	C22—C24—H24C	109.5
H12A—C12—H12B	109.5	H24A—C24—H24C	109.5
C10—C12—H12C	109.5	H24B—C24—H24C	109.5
			
C8—O1—C1—O2	−178.89 (19)	C20—O4—C13—O5	179.9 (2)
C8—O1—C1—C2	0.9 (2)	C20—O4—C13—C14	−0.9 (2)
O2—C1—C2—O3	−0.7 (3)	O5—C13—C14—O6	2.5 (4)
O1—C1—C2—O3	179.55 (17)	O4—C13—C14—O6	−176.68 (17)
O2—C1—C2—C3	178.7 (2)	O5—C13—C14—C15	179.3 (2)
O1—C1—C2—C3	−1.0 (2)	O4—C13—C14—C15	0.2 (2)
O3—C2—C3—C8	−179.90 (19)	O6—C14—C15—C20	177.15 (18)
C1—C2—C3—C8	0.7 (2)	C13—C14—C15—C20	0.6 (2)
O3—C2—C3—C4	3.4 (4)	O6—C14—C15—C16	0.2 (4)
C1—C2—C3—C4	−176.0 (2)	C13—C14—C15—C16	−176.3 (2)
C2—C3—C4—C9	−31.2 (3)	C14—C15—C16—C21	−29.2 (3)
C8—C3—C4—C9	152.4 (2)	C20—C15—C16—C21	154.17 (19)
C2—C3—C4—C5	−156.0 (2)	C14—C15—C16—C17	−154.2 (2)
C8—C3—C4—C5	27.6 (2)	C20—C15—C16—C17	29.1 (2)
C3—C4—C5—C6	−52.7 (2)	C15—C16—C17—C18	−52.6 (3)
C9—C4—C5—C6	−177.98 (19)	C21—C16—C17—C18	−178.0 (2)
C4—C5—C6—C7	52.7 (3)	C16—C17—C18—C19	51.4 (3)
C5—C6—C7—C8	−24.0 (3)	C17—C18—C19—C20	−22.8 (3)
C5—C6—C7—C10	157.55 (19)	C17—C18—C19—C22	157.9 (2)
C10—C7—C8—O1	−0.9 (3)	C22—C19—C20—O4	−1.0 (3)
C6—C7—C8—O1	−179.27 (17)	C18—C19—C20—O4	179.77 (18)
C10—C7—C8—C3	176.57 (19)	C22—C19—C20—C15	177.74 (19)
C6—C7—C8—C3	−1.8 (3)	C18—C19—C20—C15	−1.5 (3)
C1—O1—C8—C7	177.38 (19)	C13—O4—C20—C19	−179.81 (19)
C1—O1—C8—C3	−0.4 (2)	C13—O4—C20—C15	1.3 (2)
C2—C3—C8—C7	−177.94 (19)	C14—C15—C20—C19	179.97 (19)
C4—C3—C8—C7	−0.6 (3)	C16—C15—C20—C19	−2.5 (3)
C2—C3—C8—O1	−0.2 (2)	C14—C15—C20—O4	−1.2 (2)
C4—C3—C8—O1	177.12 (17)	C16—C15—C20—O4	176.29 (16)
C8—C7—C10—C12	116.2 (2)	C20—C19—C22—C23	−132.3 (2)
C6—C7—C10—C12	−65.5 (3)	C18—C19—C22—C23	46.9 (3)
C8—C7—C10—C11	−120.9 (2)	C20—C19—C22—C24	104.0 (3)
C6—C7—C10—C11	57.4 (3)	C18—C19—C22—C24	−76.8 (3)

### Hydrogen-bond geometry (Å, º)

**Table:**

*D*—H···*A*	*D*—H	H···*A*	*D*···*A*	*D*—H···*A*
O3—H3···O5^i^	0.87 (3)	1.81 (3)	2.627 (2)	157 (3)
O6—H6···O2^ii^	0.84 (3)	1.93 (3)	2.727 (2)	156 (3)

Symmetry codes: (i) −*x*+2, *y*−1/2, −*z*+2; (ii) −*x*+2, *y*+1/2, −*z*+2.
